# Monitoring the physical and insecticidal durability of the long-lasting insecticidal net DawaPlus^®^ 2.0 in three States in Nigeria

**DOI:** 10.1186/s12936-020-03194-9

**Published:** 2020-03-30

**Authors:** Emmanuel Obi, Festus Okoh, Sean Blaufuss, Bolanle Olapeju, Joel Akilah, Okefu Oyale Okoko, Abidemi Okechukwu, Mark Maire, Kehinda Popoola, Muhammad Abdullahi Yahaya, Chigozie Jesse Uneke, Samson Awolola, Olivier Pigeon, Stella Babalola, Hannah Koenker, Albert Kilian

**Affiliations:** 1 PMI VectorWorks Project, Tropical Health LLP, Abuja, Nigeria; 2 National Malaria Elimination Programme, Abuja, Nigeria; 3 PMI VectorWorks Project, JHU Center for Communication Programs, Baltimore, MD USA; 4 U.S. President’s Malaria Initiative, U.S. Agency for International Development, Abuja, Nigeria; 5 U.S. President’s Malaria Initiative, Centers for Disease Control and Prevention, Abuja, Nigeria; 6 Dept. of Zoology, University of Ibadan, Ibadan, Nigeria; 7 Dept. of Biological Sciences, Usmanu Danfodiyo University, Sokoto, Nigeria; 8 Dept. of Medical Microbiology/Parasitology, Ebonyi State University, Abakaliki, Nigeria; 9 Nigerian Institute for Medical Research, Lagos, Nigeria; 10 Walloon Agricultural Research Centre, Gembloux, Belgium; 11 PMI VectorWorks Project, Tropical Health LLP, Montagut, Spain

**Keywords:** LLIN durability, Monitoring, Nigeria

## Abstract

**Background:**

Following guidance from the US President’s Malaria Initiative, durability monitoring of DawaPlus^®^ 2.0 brand of long-lasting insecticidal net (LLIN) distributed during the 2015/16 mass campaign was set up in three ecologically different states: Zamfara, Ebonyi and Oyo.

**Methods:**

This was a prospective cohort study of representative samples of households from each location, recruited at baseline, 1 to 6 months after the mass campaign. All campaign nets in the households were labelled and followed up over a period of 36 months in Zamfara and Ebonyi and 24 months in Oyo. Primary outcome was the “proportion of nets surviving in serviceable condition” based on attrition and integrity measures and the median survival in years. The outcome for insecticidal durability was determined by bio-assay from sub-samples of campaign nets.

**Results:**

A total of 439 households (98% of target) and 1096 campaign nets (106%) were included in the study. Definite outcomes could be determined for 92% of the cohort nets in Zamfara, 88% in Ebonyi and 75% in Oyo. All-cause attrition was highest in Oyo with 47% no longer present after 24 months, 53% in Ebonyi and 28% in Zamfara after 36 months. Overall only 1% of all campaign nets were used for other purposes. Estimated survival in serviceable condition of the campaign nets was 80% in Zamfara, 55% in Ebonyi (36 months follow-up) and 75% in Oyo (24 months follow-up) corresponding to median survival of 5.3, 3.3, 3.2 years, respectively. Factors associated with better survival were exposure to social messaging combined with a positive net-care attitude and only adult users. Failing to fold the net when hanging and having children under 5 years of age in the household negatively impacted net survival. Insecticidal effectiveness testing at final survey showed knock-down rates of 50–69%, but 24-h mortality above 95% resulting in 100% optimal performance in Ebonyi and Oyo and 97% in Zamfara.

**Conclusions:**

Results confirm the strong influence of net-use environment and behavioural factors in the physical survival of the same LLIN brand, which can increase the time until 50% of nets are no longer serviceable by up to 2 years.

## Background

Malaria prevention with long-lasting insecticidal nets (LLINs) has seen a tremendous scale-up in Africa south of the Sahara in the last decade. Many countries have achieved high ownership coverage with LLINs following mass distribution campaigns, as well as continuous distribution strategies, and are approaching the target of at least 80% LLIN access for the population at risk as recommended by the World Health Organization (WHO) [1]. A critical question now is how to sustain access. To do so, it is important to understand how long on average a distributed LLIN remains in the household and continues to protect net users, termed ‘durability’. This information is useful for deciding how frequently LLINs need to be replaced and to identify product(s) that perform poorly in a specific environment.

LLIN durability has two components: the insecticidal durability/effectiveness and the physical durability. Guidance for how to assess insecticidal effectiveness has been well established by WHO since 2005 [2]. Physical durability in turn comprises the loss of nets (attrition) and the physical condition of surviving nets (integrity). Recognition that damage to nets could impact on their usefulness was published as early as 1982 [3], but attempts to capture the level of damage in early studies were poorly standardized, did not provide an overall metric of damage, and did not allow direct comparison of results [4–6]. A first suggestion of a more standardized approach was made in 2008 in the form of a Hole Index with three defined hole size categories [7] analogous to the spleen index well established in malariology. This was then extended to a proportionate Hole Index (pHI) that took into account the relative size of hole categories in 2011 [8]. Finally, further refinement of the pHI, done in the context of the WHO Vector Control Technical Expert Group, established a cut-off level of damage at which nets are no longer considered serviceable [9]. With this comprehensive guidance now available [10, 11], the WHO recommends that all malaria control programmes that distribute LLINs should also routinely monitor their durability [10], ideally using a prospective study design. Other donors and implementation partners, such as the US President’s Malaria Initiative (PMI) have taken up this recommendation and also encourage routine monitoring of LLIN durability in the countries it supports.

LLIN durability monitoring in Nigeria is one of the priorities of the Integrated Vector Management Sub-committee of the National Malaria Elimination Programme. Assessment of physical durability started in Nigeria in 2012 in the context of the PMI-funded NetWorks project. In three different eco-geographical zones a 3-year, retrospective study monitoring the physical survival of campaign nets (DawaPlus^®^ 2.0, 100-denier polyester LLIN) was carried out. Sites were located in Zamfara, Nasarawa and Cross River States. The study showed a significant variation in median survival between the sites of between 3.0 and 4.7 years which were driven mainly by differences in attitudes and behaviour of the households [12]. This has been further confirmed by another study where behaviour change communication was able to significantly improve household attitudes towards care and repair, which resulted in better physical condition of nets in these households and an estimated 9-month extension of net survival [13].

The objectives of the present study were to: (i) monitor physical and insecticidal durability of the same LLIN brand (DawaPlus^®^ 2.0) in different net-use environments in Nigeria using a prospective cohort design; and, (ii) identify major determinants influencing LLIN durability.

## Methods

### Study sites

**Fig. 1:**
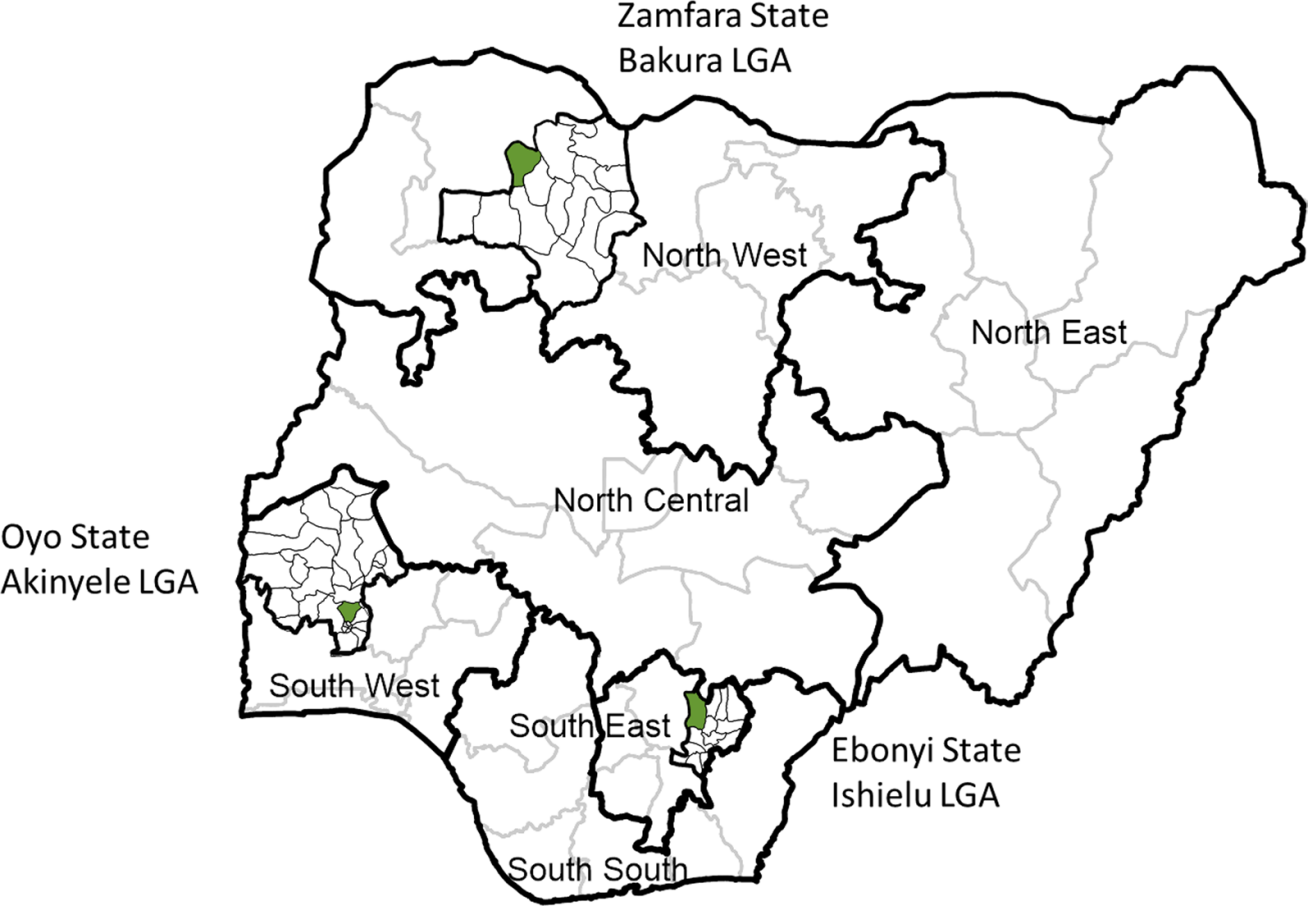
Location of study sites within Nigeria

Three sites were selected purposively with the National and State Malaria Elimination Programmes (NMEP and SMEP) representing different parts of the country with differing ecological and socio-demographic environments. Priority criteria were having the same brand of LLIN during the most recent mass campaign and having an entomological surveillance site nearby. In each of the selected states one Local Government Area (LGA) was purposively selected as the study site. The locations are shown in Fig. 1 and can be described briefly as follows:

Zamfara (Bakura LGA) is situated in the northwest geopolitical zone and belongs to the Sudan savannah ecological zone. Climate is tropically warm with temperature rising to 38 °C and above between March and May. Rainy season starts in late May to September while the ‘cold’ season, known as Harmattan, lasts from December to February. This results in highly seasonal malaria transmission with meso- to hyperendemicity during the rainy season. Zamfara State is mainly populated by Hausa and Fulani people. Bakura LGA had a 2015 population of 268,944 based on the campaign registration data and the LLIN distribution campaign took place in September 2015.

Ebonyi (Ishielu LGA) is situated in the southeast geopolitical zone and belongs to the Guinea savannah ecological zone. The climate is tropical and humid and malaria transmission is perennial. The dry season lasts from November to March while the wet season starts from April and ends in October. Ebonyi State is inhabited primarily by the Igbo people. The population of Ishielu LGA was 189,512 based on the 2015 campaign registration data and the mass campaign took place in September 2015.

Oyo State (Akinyele LGA) is situated in the southwest geopolitical zone near the state capital of Ibadan and belongs to the Guinea savannah ecological zone. The climate is equatorial with dry and wet seasons similar to Ebonyi State and relatively high humidity. Malaria transmission is essentially perennial at meso-endemic levels. Oyo state is predominantly occupied by Yoruba people. The population of Akinyele LGA was 285,565 based on the 2016 campaign registration data and the campaign was carried out in August 2016 approximately 1 year after the campaigns in Zamfara and Ebonyi States.

### Study design

This was a prospective study of a representative cohort of LLINs distributed during the 2015/16 mass distribution campaigns and followed for up to 3 years. The design was based on the guidance from the PMI for LLIN durability monitoring [14] and in this case compared the durability of the same LLIN brand between the different locations. The brand was DawaPlus^®^ 2.0, a 100-denier polyester LLIN in either white or blue colour. This LLIN brand uses a coating technology with a loading dose of 80 mg/sq m of deltamethrin and obtained interim WHOPES recommendation in July 2009 [15] and WHO prequalification in January 2018 [16]. In March 2019, the prequalification was transferred to a new manufacturer (NRS Moon Netting FZE) and the product is now called Tsara Soft^®^ listed under WHO prequalification number 028-003. Within 6 months of the respective mass distribution campaigns LLINs were sampled and followed up after 12, 24 and 36 months through household surveys. Due to the delay in the Oyo LLIN campaign and a fixed end date for the study funding this site was only followed up for 24 months. At each time point measures of physical durability were assessed (attrition and integrity) using a household questionnaire and net damage assessment tools. For all data points after baseline, 30 campaign nets per site were sampled and retrieved for assessment of insecticidal effectiveness (bio-assay). Based on questionable results from bio-assay tests from Zamfara and Ebonyi at 12 months (see Table 7) a sub-sample of 10 of these nets was sent for chemical analysis of the active ingredient.

### Sample size and sampling

Sample size was calculated to detect a difference of ± 9%-points from a 50% LLIN survival point estimate after 3 years as significant at the 95% confidence level or an 18% difference between two sites with the same brand of LLIN. This is equivalent to a deviation from the assumed 3-year median survival by 10–12 months. Further assumptions were a power of 80%, design effect of 2.5, all-cause attrition of 35% and attrition due to wear and tear of 20% over 3 years [12], an initial household non-response rate of 5%, campaign distribution of one LLIN for every two people with rounding up for odd-numbered households, and an initial loss between campaign and baseline survey of 8% of the campaign nets. This resulted in the need for a cohort of 345 campaign nets to be recruited per site. Based on an estimated average household size of five persons this required 150 households sampled from 15 clusters with 10 households each.

Clusters (communities) were sampled with probability proportionate to size using the campaign registration lists as sampling frame. Households within clusters were selected using simple random sampling from lists of eligible households prepared by the field teams on the day of the survey. For communities with more than 200 households a segmentation approach was used and only the selected segment was sampled. Up to five replacement households were sampled per cluster to substitute in case a sampled household had not received nets from the campaign or did not consent to participate. Within each household all LLINs identified as being from the campaign by brand, colour, and report by the respondent were labelled with a unique ID number and barcode for follow-up, even when they were still in the package at the time of the baseline survey.

Campaign nets for bio-assay testing were sampled from the cohort (two nets per cluster) only at the final survey using simple random sampling. For the 12- and 24-months surveys campaign nets were sampled from neighbouring households as follows: within each cluster two index households were randomly identified from the cohort and when the field teams reached these households, they went left to the next neighbour that had campaign nets and consented to give them up for the study. A brief questionnaire was filled for these nets regarding use and washing. For all LLINs collected for bio-assay new replacement LLINs were given.

### Field procedures

An implementation team of nine individuals was established per site, with one overall site coordinator and two field teams each consisting of one supervisor and three interviewers. Activities in the field were overseen by staff of SMEP and NMEP. Interviewers and supervisors were selected so that they were culturally acceptable, had good knowledge of the local languages and experience in conducting household surveys.

A 5-day training was held at baseline and 3-day refresher training before each follow-up survey. Special emphasis was put on the process of a standardized assessment of net damage using a template to identify hole size categories and tallying hole counts using an application on the digital devices used for data entry. The questionnaire had three main modules: one for the household respondent, a second for the cohort campaign nets (including nets lost between campaign and baseline survey), and a third module for other nets owned by the household at each time point. In addition, a list of household members and assets was obtained at baseline and at the final survey. GPS coordinates were recorded at baseline and used to track household during follow up. If households moved within the clusters the new homes were identified, if they moved outside the cluster, they were considered lost to follow-up. The questionnaire and all other tools are publicly available (www.durabilitymonitoring.org).

The baseline survey took place in March 2016 in Zamfara and Ebonyi and December 2016 in Oyo. All follow-up surveys took place in September, in 2016 only for Zamfara and Ebonyi and in 2017 and 2018 for all three sites.

### Laboratory analysis

Bio-assays were carried out at the Nigerian Institute of Medical Research in Lagos using the standard WHO bio-assay cone test procedure [2]. A pyrethroid-susceptible Kisumu strain of *Anopheles gambiae* sensu stricto was used with five mosquitoes per cone, five sites tested on each net (4 sides and roof panel). Two tests were conducted per location on each of the panels for a total of 10 cone tests with 50 mosquitoes per net. Recorded were 60-min knock-down (KD60) and 24-h mortality and then combined as optimal insecticidal effectiveness (KD60 ≥ 95% or mortality ≥ 80%), minimal effectiveness (KD60 ≥ 75% or mortality ≥ 50%), or failure (not reaching minimal effectiveness criteria) [9].

Chemical residue analysis was done at the Walloon Agricultural Research Centre in Gembloux, Belgium. Five pieces of netting were tested per net from the same locations as for bio-assays and the fabric weight per surface area was recorded. The five samples were then cut into small pieces and pooled to get a homogeneous sample per net. Active ingredient was extracted using sonication and shaking with heptane. Active ingredient was determined by High Performance Liquid Chromatography with UV Diode Array (HPLC–DAD) according to Collaborative International Pesticides Analytical Council standard (CIPAC 333/LN/(M)/3).

### Data management

For data collection, tablet PCs (Samsung Galaxy Tab 5) were used and installed with the Open Data Kit (ODK) software for the questionnaire and Open Street Map for Android (OSMAND) for household tracking. Data from each field team were collected daily and directly uploaded to a secure data base if internet was available or collected on a local storage device by the coordinator until it could be transferred. Data were converted from ODK to comma-delimited data files using the ODK briefcase tool for inspection of incoming data and daily feedback was provided to the teams. For each survey round, updated lists were compiled from the household and cohort net master files and preloaded on the ODK system including all households and cohort nets for which no definite outcome was available to date. After completion of the surveys, datasets were transferred to Stata version 14.2 (Stata, Texas, USA) for further aggregation, consistency checks and preparation for analysis. Stata do-files (macros) developed by the PMI VectorWorks project were applied and adjusted as needed [14]. For the final analysis datasets from all four surveys were merged.

### Data analysis

#### Definition of outcomes

The primary outcome measure was the physical net survival and was defined as the proportion of cohort nets received from the LLIN campaign still in serviceable physical condition (definition provided below) [9]. For the calculation of this outcome two interim outcomes were calculated as follows:

Net attrition rate due to wear and tear was defined as the proportion of originally received nets which were reported lost due to wear and tear (thrown away, destroyed or used for other purposes) at the time of assessment. Nets received but given away for use by others, stolen or sold were excluded from the denominator. Similarly, nets with unknown outcome were excluded.

Net integrity was measured first by the proportionate Hole Index (pHI) as recommended by WHO [10]. Holes in cohort LLINs were counted and categorized into four different sizes: size 1: 0.5–2 cm, size 2: 2–10 cm, size 3: 10–25 cm, and size 4: larger than 25 cm in diameter. The pHI for each net was then calculated as the number of holes multiplied by the size category weights as suggested by WHO [10]. Based on the pHI each net was then categorized as ‘good’, ‘damaged’, ‘serviceable’, or ‘torn’ as follows [10]:

- Good: total hole surface area < 0.01 sq m or pHI < 64
- Damaged: total hole surface area 0.01–0.1 sq m or pHI 65–642
- Torn: total hole surface area > 0.1 sq m or pHI > 642
- Serviceable: total hole surface area ≤ 0.1 sq m or pHI ≤ 642 (good or damaged)

In order to be able to compare physical survival measured at different time points, the outcome of median net survival was estimated defined as the time in years until 50% of the originally distributed LLIN were no longer serviceable. Two approaches were used to estimate median survival. At each time point the proportion surviving in serviceable condition were plotted against the hypothetical survival curves with defined median survival [9] (Additional file 1) and the median survival was taken as the interpolated position of the data point on a horizontal line between the two adjacent median survival curves. After the final survey median net survival was calculated from at the last two time points provided, both were below 85% (when the hypothetical curves are linear), using the following formula:

$${\text{tm}} = {\text{t}}1 + \frac{{\left( {{\text{t}}2 - {\text{t}}1} \right)*\left( {{\text{p}}1 - 50} \right)}}{{\left( {{\text{p}}1 - {\text{p}}2} \right)}},$$

where tm is the median survival time, t1 and t2 the first and second time points in years and p1 and p2 the proportion surviving to first and second time point respectively in per cent. Confidence intervals for this estimate were calculated by projecting the 95% CI from the survival estimates in the same way as described above.

### Explanatory variable preparation

Overall household attitudes towards net care and repair were measured using a set of Likert score questions where a statement was read to the respondent (head of household or spouse) and the level of agreement recorded. These were analysed by recoding the four-level Likert scale score to have a value of − 2 for ‘strongly disagree’, − 1 for ‘disagree’, + 1 for ‘agree’ and + 2 for ‘strongly agree’. These attitude scores for each respondent were then summed and divided by the number of statements to calculate an average household attitude score for which 0 represents a neutral result and positive values a positive result. For each site the proportion of households with a score above 1.0 (very positive attitude) were calculated at each survey.

Further aggregation of results was done across all four surveys. For household and net risk factors for durability the following categories were used: ‘never’ = responded with ‘never’ in all surveys the household participated; ‘at times’ = household reported the behaviour as ‘sometimes’ in at least one survey round or had conflicting statements; ‘always’ = responded with ‘always’ in all surveys the household participated. Exposure and attitude were similarly aggregated, i.e., ‘once’ = reported exposure or positive attitude score at one of the four survey rounds; ‘twice or more’ = at two or more survey rounds.

A wealth index was calculated for the baseline data set using the basic household assets and a principal component analysis with the first component used as the index. Households were then grouped into tertiles. The full household data collection and wealth index was repeated at the final survey. However, at the 12 and 24 months no specific household or member data were collected.

### Statistical analysis

For continuous variables, arithmetic means were used to describe the central tendency and the t-test for comparison of groups for normally distributed data. Otherwise, median and Kruskal–Wallis test were used. Proportions were compared by contingency tables and the Chi-squared test used to test for differences in proportions. For calculation of confidence intervals around estimates, the intra- and between-cluster correlation has been taken into account by applying the *svy* command in Stata.

Data were set up for survival analysis as a duration format dataset where each time interval for a net is a separate observation. Analysis was done using an intention-to-treat approach, i.e., risk of failure was considered to start at the day of distribution irrespective of whether or when the net was hung and used. Failure was defined as a net being lost to wear and tear or torn based on physical assessment (pHI). The time of failure was directly calculated from the report of time of loss by the respondent or taken as the mid-point between the last two surveys if unknown. A secondary analysis used a per-protocol approach where the risk of damage was considered to begin only when a net was first found hanging. Basic survival analysis was done using Kaplan–Meier estimations of survival function. Determinants of survival were explored using Cox proportionate hazard models. Separate models were constructed for household factors and for net level factors, such that models with net-level factors included only nets that had been ever hung for use during the study. Factors were tested first in individual models which were then used to construct the final multivariable models. Final model fit was tested using a linktest and Schoenfeld residuals and log–log plots were used to check the proportionate hazard assumption.

## Results

### Sample characteristics

At baseline a total of 439 households were recruited representing 98% of the target and 1096 campaign nets labelled for follow up (106% of target). The follow-up status of households and cohort nets is shown in Table 1. The most common reason for loss to follow-up for households was that they lost all their cohort nets and this varied between 19% in Oyo and 9% in Zamfara. The second most common reason was moving away outside the sampled community and this again was most common in Oyo with 16% and around 5% in the other sites. Only one household in Oyo refused participation. At the final survey 132 households participated in Zamfara (89% of recruited), 117 in Ebonyi (81%) and 109 in Oyo (74%). By the end of the study a definite outcome was determined for 92% of the cohort nets in Zamfara, 88% in Ebonyi and 75% in Oyo. The lower rate in Oyo was driven by the higher mobility of the population and using nets in other locations such as boarding schools.

**Table 1:** Follow-up status of recruited households and campaign cohort nets after final survey

Variable	Zamfara	Ebonyi	Oyo^a^
	N = 148	N = 144	N = 147
Households			
Still has any campaign nets	84.5%	73.6%	63.5%
Lost all their campaign nets	9.4%	16.6%	18.9%
Moved away	5.4%	5.6%	15.5%
Refused	0.0%	0.0%	0.7%
Nobody home at survey	0.7%	4.2%	1.4%

	N = 357	N = 367	N = 372
Campaign cohort nets			
Known outcome	91.6%	88.0%	74.7%
Unknown outcome	8.4%	12.0%	25.3%
Household moved away or refused	5.0%	4.4%	15.1%
Net used elsewhere	0.6%	0.0%	6.2%
Fate of net unknown	2.8%	7.6%	4.0%

^a^At 24 months of follow-up

The demographic and socio-economic characteristics of the sampled households in the three sites showed some similarities but more differences and details are shown in Additional file 2. Mean household size was 5.0 in Ebonyi, 5.5 in Zamfara and 4.4 in Oyo. Very few households were headed by women, 2% in Zamfara, 8% in Ebonyi and 19% in Oyo. Educational status of male heads of households was poorest in Zamfara with 41% non-literate and only 18% attending any secondary education, followed by Ebonyi with 35 and 49%, respectively, and best in Oyo with 30 and 55%, respectively. Female head of household’s education in Oyo was significantly lower with 60% non-literate and 20% with secondary education (p = 0.03).

The Ebonyi and Zamfara sites were both rural and predominantly agricultural while the Oyo site had more peri-urban characteristics and was economically better off than the other two sites. In Zamfara 91% of households had agricultural land and some livestock as an economic resource, in Ebonyi this rate was 69% with another 26% of households having one or the other. In contrast, only 41% of households in Oyo had farmland and livestock but 25% of households had non-agricultural income sources compared to 4% in Ebonyi and 0.4% in Zamfara. The peri-urban characteristic of the Oyo site can also be seen in the ownership of means of any transport which was lower in Oyo, 41% compared to 83% in Ebonyi and 73% in Zamfara, but included more cars (19% of households) rather than bicycles, which were the dominant means of transport in the other sites. Ownership of household assets representative of wealth such as television, fridge or electric fan were significantly more common in Oyo (Additional file 2). In Oyo 83% of households owned at least one mobile phone compared to 66% in Zamfara and 49% in Ebonyi where some areas of the LGA did not have phone coverage. The difference in smart phones was even more striking with 31% of Oyo households owning them while they were essentially absent (< 1%) in the other two sites. Access to safe drinking water was 97% in Zamfara, 93% in Oyo but only 14% in Ebonyi where 86% of households used surface water from rivers and creeks. Access to any kind of latrine was 100% in Zamfara, 73% in Ebonyi and only 67% in Oyo, but here 60% of the latrines were improved pit latrines or flush toilets. There was no evidence of significant changes in socio-economic status during the study period.

### Risk factors of physical durability

A number of behavioural factors that are known or thought to be associated with the risk of damage to nets were monitored. These can be divided into four groups: factors of the net-use environment in the household, knowledge and attitudes towards net care and repair by the household respondent, net handling and washing, and type of sleeping place. Household-related factors are presented in Table 2 and these depended exclusively on the respondents who in Zamfara were 78% the spouse and 20% the head of household. In Ebonyi the proportions were 47% spouse and 45% head of households and in Oyo 58% and 34%, respectively. The remainder (6% on average) were other adult household members. Seeing rodents or their traces around the house was almost ubiquitous in Zamfara and Oyo throughout the study with 98 and 95%, respectively and still very common in Ebonyi with 83%. Storing food in a room used for sleeping is thought to attract rodents which in turn could damage the nets. This practice was very common in Zamfara and Oyo and less so in Ebonyi. Cooking in the room where people sleep can lead to heat damage on nets, particularly if the cooking fuel is firewood or charcoal. This practice was more or less absent in Zamfara with 98% of households never reporting it and it was rare in Oyo with 16% stating to do so at times and 2% always, but here less than 50% of households used firewood or charcoal. In Ebonyi, however, where 96% reported cooking with firewood, there was a higher risk with 48% stating to cook inside the sleeping room at times and 2% always.

**Table 2:** Net-use environment at household

Variable	Zamfara	Ebonyi	Oyo^a^	p-value for site comparison
	N = 148 % (95% CI)	N = 144 % (95% CI)	N = 147 % (95% CI)	
Households				
Storing of food in sleeping rooms				
Never	0.0 (–)	9.7 (4.7–19.0)	16.2 (9.0–27.5)	< 0.0001
At times	18.9 (8.9–35.9)	73.6 (65.9-80.3)	41.9 (32.8–51.2)	
Always	81.1 (64.1–91.1)	16.7 (9.0-28.8)	41.9 (29.4–55.5)	
Cooking in sleeping room				
Never	98.0 (94.2–99.3)	50.0 (38.3–71.7)	81.8 (69.8–89.7)	< 0.0001
At times	1.3 (0.3–5.2)	47.9 (35.9–60.2)	16.2 (8.5–28.9)	
Always	0.7 (0.1–4.9)	2.1 (0.7–6.0)	2.0 (0.5–8.4)	
Exposure to net use or care messages				
Never	0.0 (–)	19.4 (8.5–38.6)	5.4 (1.8–14.6)	0.0009
Once	2.0 (0.7–5.8)	19.4 (11.1–31.8)	22.3 (14.6–32.5)	
Twice or more	98.0 (94.2–99.3)	61.1 (38.1–80.1)	72.3 (59.8–82.1)	
Very positive net care attitude (score > 1.0)				
Never	0.0 (–)	57.6 (40.0–73.5)	39.2 (25.3–55.1)	< 0.0001
Once	3.7 (1.6–7.1)	36.1 (24.1–50.1)	35.1 (23.8–48.5)	
Twice or more	96.6 (92.9–98.4)	6.2 (2.3–15.9)	25.7 (17.0–36.7)	

Results were aggregated across all four surveys i.e. ‘never’ = responded with ‘never’ in all surveys the household participated; ‘at times’ = household reported the behaviour as ‘sometimes’ in at least one survey round or had conflicting statements; ‘always’ = responded with ‘always’ in all surveys the household participated. Exposure and attitude were similarly aggregated, i.e., ‘once’ = reported exposure or positive attitude score at one of the four survey rounds; ‘twice or more’ = at two or more survey rounds

^a^Only 24 months of follow-up

The two other household-level factors monitored were exposure to social and behaviour change communication (SBCC) messages on net use and care in the 6 months preceding each survey and the net care attitude scores based on a set of Likert score questions. Both are thought to induce or increase practices that protect nets from damage and they were closely correlated (p < 0.0001). Among households that had never reported any SBCC exposure throughout the study, 96% also never had a very positive net care attitude score. Conversely, 80% of households that had reported SBCC exposure at least twice were found with a very high attitude score at least once. However, a single SBCC exposure was not associated with a positive attitude score as 72% of households in this category never had a very positive attitude score. This relationship between SBCC and net care attitude was similar at all three sites but strongest in Zamfara which also had the highest results in SBCC exposure and attitude scores (Table 2). SBCC exposure was a little less intense in Oyo where only 61% of households had a very positive attitude score at least once and in Ebonyi one in five households never reported having heard or seen net related messages and 58% never had a very positive net care attitude score. The level of SBCC exposure remained relatively high 56–99% at all time points and sites during the follow-up period indicating that SBCC was not limited to the mass campaign but an ongoing activity.

SBCC channels were initially predominantly interpersonal communication (IPC) through health workers, community agents or local leaders. During the follow-up, messages through the media, mainly radio, increased at all three sites and this was most pronounced in Oyo where at the final 24-months survey 17% of SBCC exposed households had received messages only through the media and 54% from media and IPC. Messages most recalled among those exposed at all three sites were on net use (98%), hanging (96%), and caring for the net (96%). Recall of ‘ITN prevent malaria’ was also high in Zamfara (96%) and Ebonyi (85%), but lower in Oyo (69%). Lowest recall was seen for ‘repair your net’ with 94% in Zamfara, 75% in Ebonyi and only 30% in Oyo.

Durability risk factors regarding the handling and use of nets when hanging are shown in Table 3. Again, Zamfara had the highest level of potentially damage-preventing behaviours. Of the hanging cohort nets, 99% were ever found tied or folded up and out of harm’s way on the days of the survey, 39% at all observations and 60% at least some of the times. In contrast, 14% of the cohort nets in Ebonyi and 31% in Oyo were never found folded or tied. In Zamfara 93% of the cohort nets were used over bedframes, 72% over unfinished bedframes, while in Ebonyi 36% and in Oyo 69% were used over foam mattresses or reed mats without bedframes. In Zamfara a lower proportion was used by children alone while in Oyo use of cohort nets by adults only was highest with 54%.

**Table 3:** Net-use environment and washing of cohort nets from campaign

Variable	Zamfara	Ebonyi	Oyo^a^	p-value for site comparison
	N = 357 % (95% CI)	N = 367 % (95% CI)	N = 372 % (95% CI)	
Cohort nets				
Ever found hanging	99.4 (96.1–99.9)	85.3 (79.6–89.5)	55.1 (48.3–61.7)	< 0.0001
Ever used	98.9 (95.9–99.7)	80.4 (73.6–85.7)	60.5 (52.4–68.0)	< 0.0001

	N = 355	N = 313	N = 205	
Cohort nets ever hung				
Tied up or folded when hanging				
Never	0.8 (0.2–3.5)	14.4 (8.6–23.1)	31.2 (19.3–46.3)	< 0.0001
At times	59.7 (43.2–74.3)	39.9 (30.1–50.6)	16.6 (10.7–24.9)	
Always	39.4 (24.8–56.3)	45.7 (33.4–58.5)	52.2 (34.9–69.0)	
Type of sleeping place^b^				
Bed frame (finished)	20.7 (12.8–31.8)	9.3 (3.7–21.3)	6.3 (3.1–12.6)	< 0.0001
Bed frame (sticks)	72.2 (59.8–81.9)	54.5 (46.2–62.5)	10.2 (6.7–15.3)	
Foam mattress	3.1 (1.6–6.0)	35.6 (27.4–44.6)	62.9 (51.3–73.3)	
Reed mat	4.0 (2.1–7.5)	0.6 (0.2–2.3)	20.5 (11.2–34.5)	

	N = 353	N = 295	N = 225	
Cohort nets ever used				
Dominant user group				
Children only	2.6 (1.1–6.2)	8.1 (5.8–11.3)	9.4 (4.2–19.5)	< 0.0001
Children with adults	73.9 (65.3–80.9)	63.7 (59.8–67.4)	37.1 (28.4–46.7)	
Adults only	23.5 (16.9–31.8)	28.2 (23.7–33.0)	53.6 (43.8–63.0)	
Ever washed	98.0 (95.2–99.2)	53.9 (32.9–73.6)	70.2 (55.8–81.5)	< 0.0001

	N = 347	N = 162	N = 177	
Cohort nets ever washed				
Washes last 6 months^c^				
Median (IQR)	3.3 (2.5–4.3)	2.0 (2.0–2.3)	2.0 (1.5–3.0)	< 0.0001
Use of detergent				
Never	28.0 (17.7–41.1)	98.8 (91.7–99.8)	51.4 (38.2–64.4)	< 0.0001
At times	60.8 (52.6–68.5)	1.2 (0.2–8.3)	17.5 (11.8–25.2)	
Always	11.2 (6.5–18.8)	0.0 (–)	31.1 (20.0–44.8)	
Drying net outside				
Never	0.6 (0.1–2.2)	67.3 (48.8–81.6)	3.4 (1.2–9.1)	< 0.0001
At times	8.1 (2.5–23.5)	13.0 (5.0–29.6)	8.5 (4.4–15.8)	
Always	91.3 (74.8–97.4)	19.7 (8.1–40.7)	88.1 (77.2–94.2)	
Drying over bush or fence				
Never	30.0 (19.8–42.7)	99.4 (95.7–99.9)	66.7 (48.0–81.3)	< 0.0001
At times	60.2 (51.5–68.4)	0.6 (0.1–4.3)	13.6 (7.0–24.6)	
Always	9.8 (5.1–34.3)	0.0 (–)	19.8 (10.4–34.3)	

^a^Only 24 months of follow-up

^b^Lowest type of sleeping place ever reported for net

^c^Average of all recorded 6 months episodes for each net

Table 3 also presents the data on washing and drying of the cohort nets. Washing frequency in the previous 6 months was highest in Zamfara with an estimated 6–7 washes per year compared to four in the other two sites. The lowest use of a commercial detergent, compared to the sometimes milder country soaps, was seen in Ebonyi followed by Zamfara and Oyo where almost one-third of cohort nets were always washed with a detergent. In Zamfara and Oyo nets were dried almost exclusively outside while in Ebonyi two-thirds of the cohort nets were always dried inside. Finally, drying nets on bushes or fences which potentially can cause damage was most common as a regular practice in Oyo, but also seen at times in Zamfara.

**Fig. 2:**
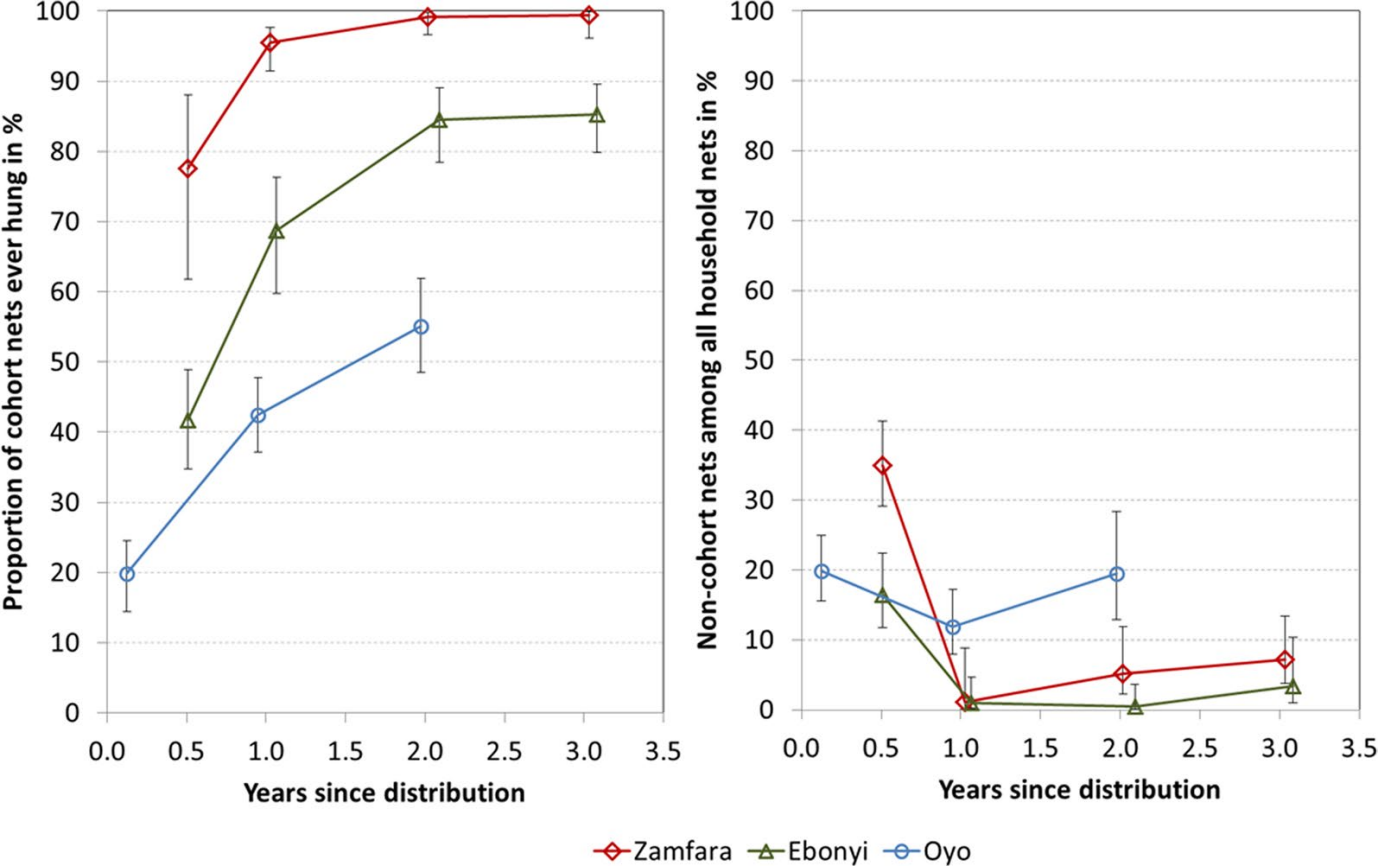
Cohort nets ever found hanging (left) and share of non-cohort nets among household net crop (right)

Almost all of the cohort nets in Zamfara (99%) were found hanging at least once in the course of the study (Table 3) and even at baseline already 78% were found hanging increasing to 96% ever hung after 12 months (Fig. 2, left panel). Hanging of cohort nets in Ebonyi was still high with 85% but here it took 24 months until the baseline level of Zamfara was reached. In contrast, hanging of cohort nets in Oyo started out at 20%, one and a half months after distribution but only reached a maximum of 55% after 2 years. From a survival analysis, with first hanging of the cohort nets as the defined outcome, estimated time until 70% of nets were hanging was 6 months in Zamfara, 12 months in Ebonyi and over 24 months in Oyo. Initially there were nets from other sources at all three sites varying between 30% of households with any non-cohort nets in Ebonyi, 46% in Oyo and 69% in Zamfara and these nets represented 17, 20 and 35% of all nets owned by the households, respectively (Fig. 2 right panel). Particularly in Oyo, there was some level of oversupply of LLINs from the campaign. The ratio of campaign LLINs per de-jure, i.e. usual household members in Oyo was 0.64 or 1.28 LLINs for every two people while this was 1.03 LLINs in Ebonyi and 0.82 LLINs in Zamfara. Furthermore, 39% of households in Oyo had received more LLINs than they should have based on the campaign distribution algorithm (one LLIN for two people rounding up for uneven numbered households). Adding the non-cohort nets, households in Oyo at baseline had 1.57 nets per every two people compared to 1.29 in Ebonyi and 1.08 in Zamfara.

At the 12-months assessment most of the non-cohort nets had disappeared. This was most pronounced in Zamfara and Ebonyi (Fig. 2, right panel). Only 16 households in Zamfara reported receiving any new nets from other sources after the campaign, which totalled 19 nets at the final survey. These were mostly from ante-natal care (ANC) or expanded programme on immunization (EPI) services at health facilities, with some also received from family and friends and a few from markets. In Ebonyi only seven households received any additional nets and only six were present at the final survey, all from the public sector. The influx of new nets was significantly higher in Oyo where 16% of households reported having received new nets during the course of the study, half from routine distribution, half from informal sources such as family and friends.

Use of cohort nets the previous night closely followed the hanging rate, i.e., if a net was hung, it was also used (Table 3). In Oyo the use rate was even higher than the hanging rate, suggesting that some nets that were consistently taken down during the day, and hence never observed hanging, were also used at night. Regular use the previous week as reported by the household respondent was high in Zamfara with 70% or higher at all time points but it must be taken into account that all surveys were conducted either at the beginning (baseline) or towards the end of the rains. Regular use of the cohort nets was far less in Ebonyi and Oyo with half or less of the nets reported as used every day of the previous week. While in Oyo over 90% of households claimed to be equally using nets in dry and rainy season, there was an increasing number of households in Ebonyi that admitted they used the nets mainly during the rains: 2% at 12 months, 21% at 24 months and 70% at 36 months. Interestingly, 95% of household respondents in Zamfara consistently reported using nets equally in the rainy and dry season.

### Attrition

**Fig. 3:**
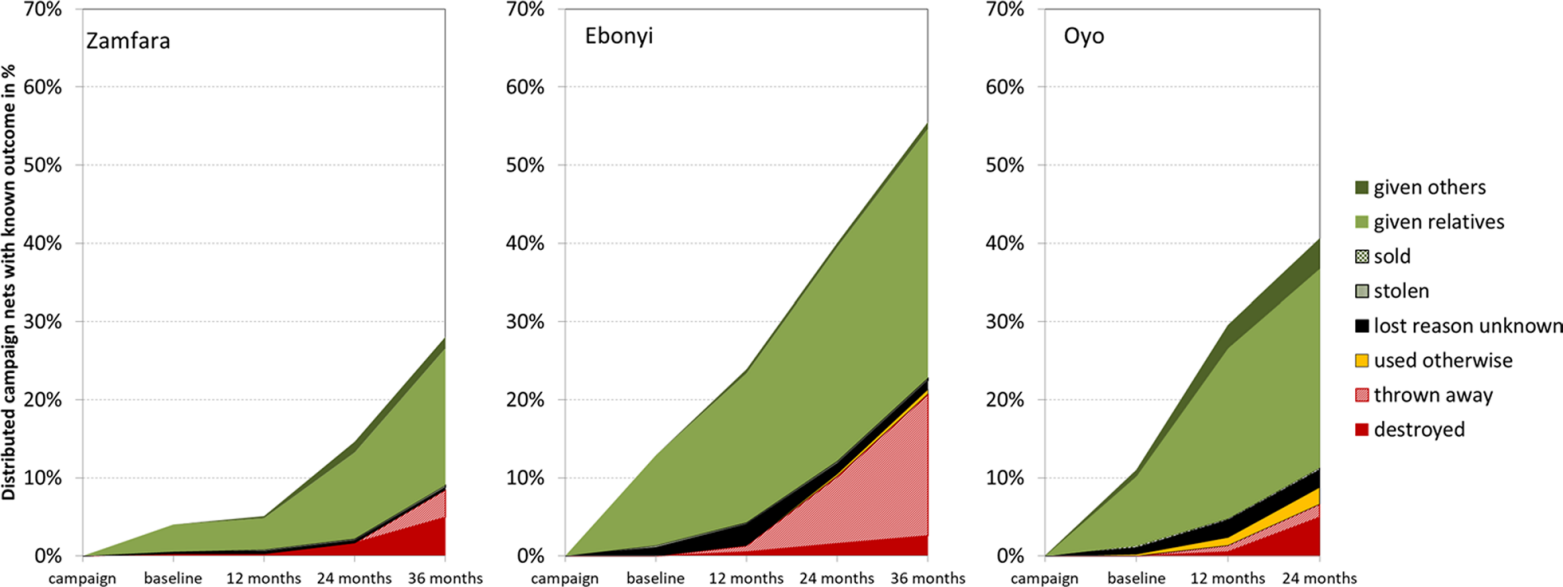
Attrition of cohort nets and their causes

All-cause attrition, i.e., the loss for any reason among cohort nets with a definite outcome (see Table 1) at the end of the study was 28% in Zamfara, 53% in Ebonyi and 47% in Oyo (after only 24 months). More importantly, attrition due to wear and tear (destroying, throwing away or use for other purposes) at the final data point was only 9% in Zamfara and 20% in Ebonyi after 36 months. In Oyo the rate was 22% after 24 months compared to 26% in Ebonyi and 12% in Zamfara at the same time point. Details of the reasons for loss over time are shown in Fig. 3. The proportion of losses due to wear and tear among all losses was very small at baseline (0% Ebonyi, 7% Zamfara, and 2% Oyo) meaning that almost all losses were due to nets being given away. The proportion gradually increased and at the final survey 38% of attrition was due to wear and tear in Ebonyi, 44% in Zamfara and 22% in Oyo (24 months only). What was done with old nets differed between sites (p = 0.003). In Ebonyi 86% of discarded nets were thrown away, 12% destroyed and 2% used for other purposes (window and door cover). In Zamfara 59% were destroyed, 41% thrown away and none used for other purposes. In Oyo the major way of discarding was destroying them (58%), but 18% were thrown away and 24% were used for other purposes (87% window and door cover, 13% protection of crops). However, repurposing nets occurred only for 2.5% of all campaign nets distributed and for which a definite outcome was available in Oyo, 0.5% in Ebonyi and none at all in Zamfara.

### Integrity

As one would expect, the proportion of cohort nets still present in the surveyed households with any sign of damage increased continuously over time reaching 89% in Ebonyi and Zamfara after 36 months (Table 4). In Oyo the rate was somewhat lower with 40% after 24 months. This is reflected in the relatively high proportion of nets in good condition after 24 months which was 77% in Oyo compared to 64% in Zamfara and 55% in Ebonyi at the same time point. However, those nets in Oyo with any holes had more damage than those in Zamfara with 11% torn and a median proportionate pHI of 189. Even after 36 months 10% in Zamfara were torn and the median pHI was 129. At the same time point 78% of surviving cohort nets in Ebonyi were in serviceable physical condition compared to 90% in Zamfara and 89% in Oyo after 24 months.

**Table 4:** Integrity of campaign nets present in households

Variable	Baseline	12 months	24 months	36 months
	% (95% CI)	% (95% CI)	% (95% CI)	% (95% CI)
	N = 357	N = 353	N = 309	N = 246
Zamfara				
Mean months since campaign	6.2	12.3	24.2	36.4
Net has any hole	14.3 (8.1–23.9)	47.0 (38.9–55.3)	73.8 (65.3–80.8)	89.0 (74.4–95.8)
Physical condition (pHI)				
Good (0–64)	97.8 (94.1–99.2)	87.0 (81.6–90.9)	64.1 (57.5–70.2)	44.7 (36.3–53.5)
Damaged (65–642)	2.2 (0.8–5.9)	11.0 (7.3–16.4)	29.5 (23.8–35.8)	45.1 (37.0–53.5)
Torn (> 642)	0.0 (–)	2.0 (1.0–4.0)	6.5 (2.9–13.8)	10.2 (6.2–15.7)
Serviceable (0–642)	100 (–)	98.0 (96.0–99.0)	93.5 (86.2–97.1)	89.8 (84.3–93.6)
Median pHI if any hole (IQR)	23 (2–40)	28 (4–76)	58 (24–189)	129 (30–376)
Has any repairs if any hole	2.0 (0.2–14.0)	1.8 (0.4–7.9)	7.5 (3.2–16.5)	13.7 (9.5–19.5)

	N = 367	N = 269	N = 232	N = 175
Ebonyi				
Mean months since campaign	6.1	12.8	25.2	37.0
Net has any hole	12.8 (8.0–19.8)	33.1 (26.1–40.9)	73.3 (67.2–78.6)	89.1 (80.6–94.2)
Physical condition (pHI)				
Good (0–64)	96.2 (92.3–98.2)	87.2 (81.7–98.2)	55.2 (46.1–63.9)	36.0 (27.2–45.9)
Damaged (65–642)	3.5 (1.7–7.2)	10.5 (6.5–16.4)	33.6 (26.5–41.6)	41.7 (32.7–51.4)
Torn (> 642)	0.3 (0.1–2.2)	2.3 (1.2–4.6)	11.2 (7.4–16.6)	22.3 (14.9–31.9)
Serviceable (0–642)	99.7 (97.8–99.9)	97.6 (95.4–98.8)	88.8 (83.4–92.6)	77.7 (68.1–85.1)
Median pHI if any hole (IQR)	29 (3–77)	33 (11–196)	104 (24–347)	213 (52–652)
Has any repairs if any hole	0.0 (–)	11.2 (5.6–25.0)	47.1 (26.2–69.1)	46.8 (27.2–67.4)

	N = 372	N = 216	N = 169	n.a.
Oyo				
Mean months since campaign	1.5	11.4	23.7	n.a.
Net has any hole	1.6 (0.5–4.6)	27.8 (19.0–38.6)	40.2 (27.7–54.3)	n.a.
Physical condition (pHI)				
Good (0–64)	99.5 (97.6–99.9)	85.2 (76.1–91.2)	76.9 (64.8–85.8)	n.a.
Damaged (65–642)	0.5 (0.1–2.4)	10.6 (5.9–18.5)	11.8 (7.4–18.4)	
Torn (> 642)	0.0 (–)	4.2 (1.6–10.4)	11.2 (4.4–25.8)	
Serviceable (0–642)	100 (–)	95.8 (89.7–98.4)	88.8 (74.2–95.6	
Median pHI if any hole (IQR)	12 (1–69)	133 (23–358)	186 (25–680)	n.a.
Has any repairs if any hole	n.a.	21.7 (10.5–39.6)	20.6 (9.8–38.4)	n.a.

Repair of nets with any holes was highest in Ebonyi with 47%, followed by Oyo with 21% and 14% in Zamfara. Female headed households tended to have a higher rate of repair if the net had any damage (40 *vs* 30%) but due to the small number of female-headed households this evidence was not very strong (p = 0.3). If repairs were made it was almost exclusively by household members in Ebonyi and Zamfara, while 20% of households in Oyo that had done any repairs said they had used a tailor. Stitching the holes up was the most common way of repair reported by 88% of repairing households followed by knotting up the holes (31%), while patching was used only by 4% (multiple responses were possible).

Damage mechanisms were explored by asking the respondents by what mechanisms their nets got holes. Across all three sites mechanical damage was consistently the most common mechanisms, but more so in Ebonyi which had a lower rate of reported rodent damage than Zamfara but a higher rate of burns than Zamfara or Oyo. The reported damage pattern at the surveys was very similar at all three sites suggesting that these results are reasonably robust.

### Survival in serviceable condition

Overall physical survival in serviceable condition, i.e., the combination of attrition due to wear and tear and the integrity of the still-existing nets was 55% in Ebonyi and 80% in Zamfara after 36 months (Table 5) and this difference was significant (p = 0.005). Survival in Oyo was 75% after 24 months, i.e., almost the same as in Ebonyi after 24 months. Survival of the campaign nets also showed a strong intra-cluster correlation, meaning that within a village it was more consistent than between villages or, in other words, net-use environment and net care and repair behaviour was similar within communities, but some were better than others. This can be inferred from the higher than expected statistical ‘design effect’ of LLIN survival, which was 4.3 in Ebonyi, 3.1 in Zamfara and 4.2 in Oyo compared to the expected 2.5.

**Table 5:** Estimated median survival in serviceable physical condition

Variable	12 months	24 months	36 months
Zamfara			
% surviving in serviceable condition (95% CI)	97.7 (95.7–98.8)	91.8 (84.1–95.9)	80.4 (72.7–86.3)
Median survival in years			
Estimated from Fig. 4	5.8	5.6	5.3
Calculated from last two data points (95% CI)	–	–	5.3 (4.6–6.4)
Ebonyi			
% surviving in serviceable condition (95% CI)	96.0 (92.5–97.9)	76.3 (67.9-83.1)	54.8 (41.4-67.6)
Median survival in years			
Estimated from Fig. 4	4.1	3.3	3.3
Calculated from last two data points (95% CI)	–	–	3.3 (2.8–4.2)
Oyo			
% surviving in serviceable condition (95% CI)	92.0 (86.0–95.6)	74.6 (60.2–85.1)	n.a
Median survival in years			
Estimated from Fig. 4	2.7	3.2	n.a
Calculated from last two data points (95% CI)	–	3.2 (2.1–5.3)	

**Fig. 4:**
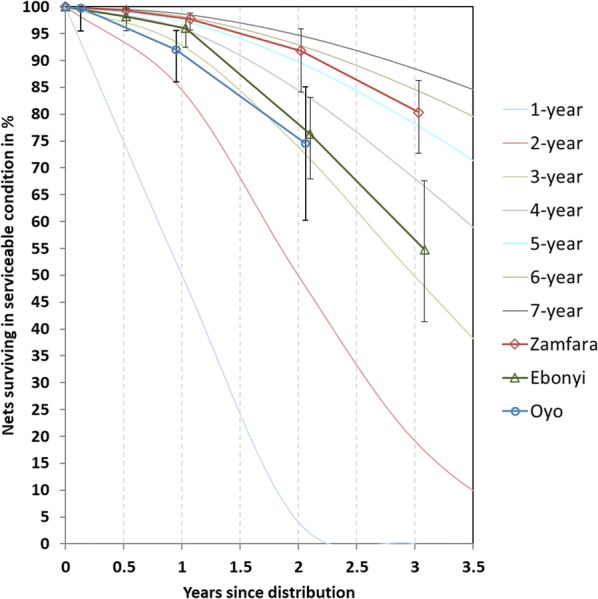
Survival of cohort nets in serviceable condition plotted against reference curves with defined median survival

When these results are plotted against the hypothetical survival curves with defined median survival (Fig. 4 and Additional file 1) it can be seen that survival roughly follows the same line in Zamfara with an estimated survival at 36 months of 5.3 years. In Ebonyi the median survival estimate dropped somewhat from around 4 years after 12 months to 3.3 years at 24 months and again 3.3 years after 36 months. Median survival estimate was lowest in Oyo but slightly increased from a median survival of 2.7 years after 12 months to 3.2 years after 24 months. Table 5 shows the median survival estimates obtained by different methods.

**Fig. 5:**
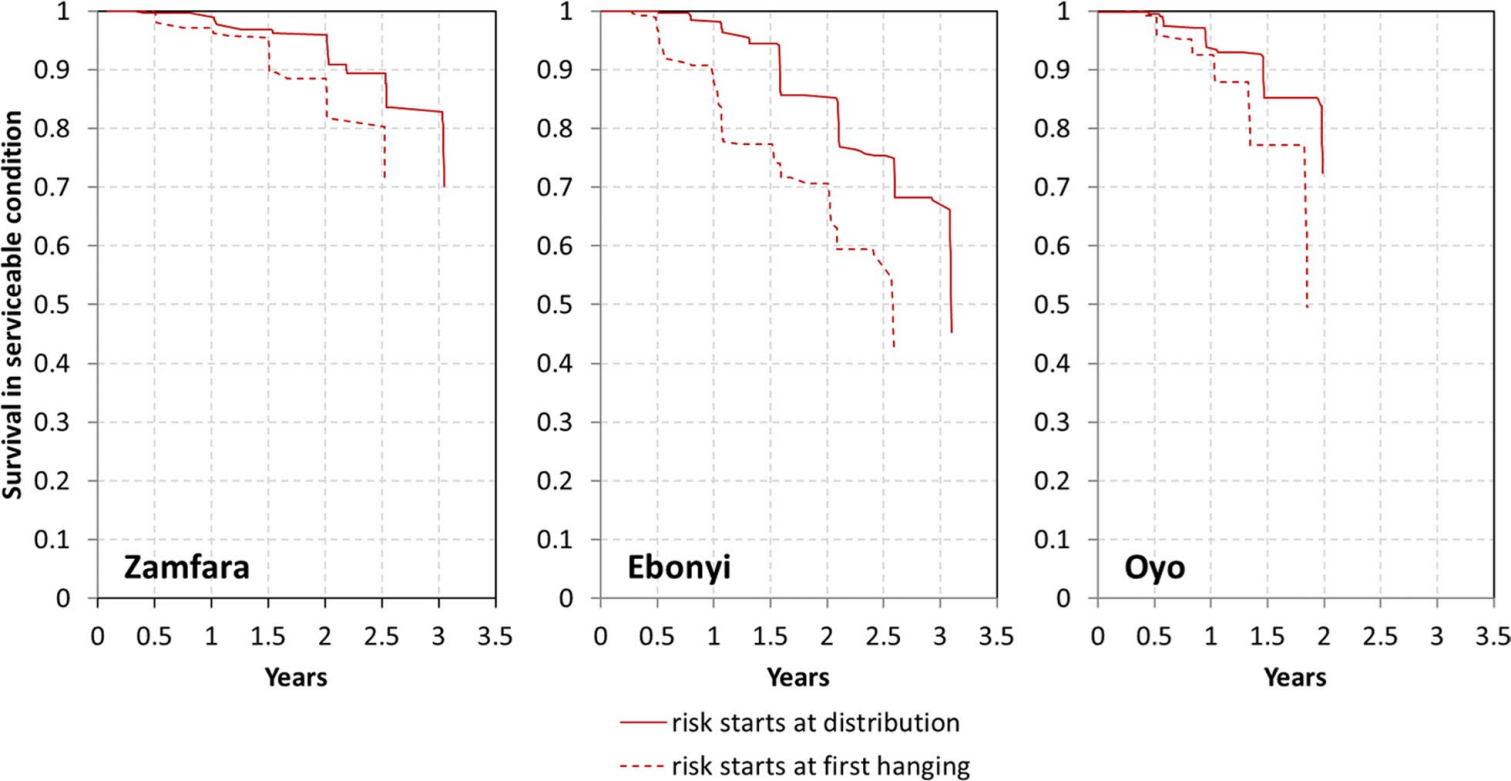
Kaplan–Meier survival functions of cohort nets comparing risk starting at distribution versus starting at first hanging

Kaplan–Meier survival curves comparing the intention to treat and per protocol analysis are shown in Fig. 5 and show a similar pattern of survival curves only shifted to the left by 0.2 to 1.0 years when risk of damage is considered to start only when the net is hung for the first time. In either analysis Zamfara had a significantly better survival (p < 0.001 in comparison with Ebonyi or Oyo). However, while the Oyo survival curve was below Ebonyi in the intention-to-treat analysis (p = 0.002) this difference disappeared in the per-protocol analysis (p = 0.5, Additional file 3).

In a bivariable Cox proportionate hazard model using the intention-to-treat approach, the hazard ratio (HR) for survival in serviceable condition in Zamfara was half that of the other two sites (HR 0.51, 95% CI 0.33–0.81, p = 0.005) reflecting the previous analysis by other methods. However, in a first multivariable Cox model, including household level variables, the difference between Ebonyi and Zamfara disappeared (Table 6) indicating that the other explanatory variables made the difference. In contrast, cohort nets in Oyo continued to have a significantly increased risk of failure suggesting that other variables not captured may have played a role here. Similarly, variables such as education of the head of household or household wealth tertile had an explanatory value in bivariable models, but not once detailed behavioural or exposure variables were included as these were closely correlated. Household-related factors that significantly improved the physical survival of cohort nets were exposure to SBCC messages at least twice combined with showing a very high attitude score at least one (aHR 0.42 compared to never exposed and never showing positive net care attitude) and never cooking inside sleeping rooms (aHR 0.43 compared to always). A negative impact factor was having any children under 5 years old at the beginning of the study which increased the hazard of failure by 38%. There was no evidence that female-headed households had a better survival of the cohort nets (aHR 0.75, 95% CI 0.41–1.38, p = 0.35).

**Table 6:** Determinants of physical durability (risk of failure to survive in serviceable condition) from Cox proportional hazard models (intention-to-treat approach)

Variable	Adjusted hazard ratio (HR)	95% CI	p-value
At household level; N = 3574 obs/1205 nets			
Site			
Ebonyi	1.00		
Zamfara	1.07	0.49–2.37	0.85
Oyo	3.50	1.52–8.09	0.004
High net care attitude score and SBCC exposure combination across surveys			
Attitude never—SBCC never	1.00		
Attitude never or once and SBCC exposure at least once	0.71	0.41–1.22	0.21
Attitude at least once—SBCC at least twice	0.42	0.22–0.80	0.009
Cooking inside the sleeping room			
Always	1.00		
At times	0.63	0.26–1.52	0.29
Never	0.43	0.19–0.95	0.037
Household had any children under 5 at baseline	1.38	0.99–1.92	0.055
At net level (nets ever hung) N = 2939 obs/982 nets			
Site			
Ebonyi	1.00		
Zamfara	0.91	0.44–1.87	0.78
Oyo	2.67	0.98–7.22	0.047
High net care attitude score and SBCC exposure combination across surveys			
Attitude never—SBCC never	1.00		
Attitude never or once and SBCC exposure at least once	0.86	0.47–1.56	0.61
Attitude at least once—SBCC at least twice	0.54	0.24–1.21	0.13
Never folding net up during day when hanging	1.99	0.94–4.25	0.072
Type of sleeping place			
Finished bedframe	1.00		
Unfinished bedframe	1.02	0.61–1.72	0.93
Foam mattress, no bedframe	1.17	0.73–1.87	0.52
Reed mat or ground	1.74	0.86–3.52	0.12
Used only by adult users	0.66	0.42–1.03	0.065
Household had any children under 5 at baseline	1.42	0.95–2.12	0.088

*obs* observations

Adding net-related variables to the model reduced the sample size by around 600 observations as this information was only available for nets ever hung. The first effect of addition of these variables was that household level determinants became weaker or disappeared (cooking in sleeping room), because it now included some of the specific behaviours that make up the positive attitude. Nonetheless, high SBCC exposure and positive care attitude still had some explanatory value (Table 6). An additional preventive factor in the final model was use of a net exclusively by adults. Negative determinants were never folding the net up when hanging, using the net over a reed mat and again having children under 5 in the household.

### Insecticidal effectiveness

Results from bioassays of the 30 campaign nets sampled at each site and time point after baseline are shown in Table 7. The samples collected at 12-months at Ebonyi and Zamfara had very poor results with only 16% and 30%, respectively, showing optimal insecticidal performance based on WHO criteria. However, results from the 24- and 36-month samples did not confirm these results. While the 60-min knock-down rates slightly declined over time, median 24-h mortality remained near 100% resulting in optimal performance in 97% of samples in Ebonyi and 98% in Zamfara. All samples showed at least minimum effectiveness. The results from the Oyo samples, which were different batches of DawaPlus^®^ 2.0, were very similar to the 24-month results of the other two sites with a median knock-down rate of 44% and median mortality of 100% corresponding to 100% optimal performance. Negative and positive controls were within expected, 0 and 100% mortality, respectively. Results from chemical residue testing from a sub-sample of the 12-months sample from Ebonyi and Zamfara showed a deltamethrin content of 1.08 g/kg and 0.83 g/kg, respectively, corresponding to 44 mg/sq m and 37 mg/sq m, respectively. This corresponds to 55% of the initial loading dose remaining in Ebonyi and 46% in Zamfara. This is lower than one would expect, but still sufficient as all except one sample had at least 15 mg/sq m deltamethrin and that sample (1.5 mg/sq m) was from Zamfara. The r-isomer of deltamethrin which tends to increase after intensive UV light exposure was only found in three of the 10 samples and in only one was it slightly elevated with 21% while the other two samples had 1 and 3%. These results do not suggest significant damage of the insecticide through storage.

**Table 7:** Results from bio-assays using WHO cone test

Variable	12 months	24 months	36 months
	N = 30	N = 30	N = 30
Zamfara			
Knock down 60 min			
Mean (95% CI)	46.8% (39.5–54.1)	66.1% (60.0–72.3)	45.2% (40.9–49.6)
Median (IQR)	50.0% (35.0–64.0)	69.0% (56.0–80.0)	43.0% (34.0–56.0)
Mortality 24 h			
Mean (95% CI)	64.4% (57.8–71.2)	99.6% (99.5–100)	98.3% (96.6–100)
Median (IQR)	70.0% (60.0–80.0)	100% (100–100)	100% (100–100)
Optimal effectiveness			
Estimate (95% CI)	30.0% (16.2–48.7)	100% (–)	96.7% (77.7–99.6)
Minimal effectiveness			
Estimate (95% CI)	80.0% (63.4–90.2)	100% (–)	100% (–)
Ebonyi			
Knock down 60 min			
Mean (95% CI)	58.3% (50.7–65.9)	52.0% (46.2–57.8)	42.3% (37.4–47.1)
Median (IQR)	60.0% (34.0–80.0)	50.0% (40.0–66.0)	40.0% (34.0–48.0)
Mortality 24 h			
Mean (95% CI)	65.0% (60.3–69.7)	99.2% (98.4–99.9)	97.3% (95.2–99.5)
Median (IQR)	64.0% (60.0–74.0)	100% (100–100)	100% (100–100)
Optimal effectiveness			
Estimate (95% CI)	16.7% (7.3–33.6)	100% (–)	96.7% (77.7–99.6)
Minimal effectiveness			
Estimate (95% CI)	90.0% (72.7–96.8)	100% (–)	100% (–)
Oyo			
Knock down 60 min			
Mean (95% CI)	53.4% (47.9–58.9)	47.5% (41.3–53.6)	n.a
Median (IQR)	54.0% (42.0–66.0)	44.0% (36.0–58.0)	
Mortality 24 h			
Mean (95% CI)	99.7% (99.2–100)	98.5% (97.0–99.9)	n.a
Median (IQR)	100% (100–100)	100% (100–100)	
Optimal effectiveness			
Estimate (95% CI)	100% (–)	100% (–)	n.a
Minimal effectiveness			
Estimate (95% CI)	100% (–)	100% (–)	n.a

## Discussion

Using a two-stage cluster sampling, prospective cohort design, this study monitored physical and insecticidal durability of one LLIN brand, the polyester-based, 100 denier DawaPlus^®^ 2.0, in three locations in Nigeria with differing net-use environment and behavioural patterns. In the rural, agricultural, northern site in Zamfara State, 80% of the cohort nets with definite outcomes survived in serviceable condition after 36 months with an estimated median survival of 5.3 years. This was significantly better than the results in the other two sites: 55% after 36 months and 3.3 years median survival in the rural, agricultural, southern site in Ebonyi State and 75% after 24 months or 3.2 years median survival in the peri-urban site in Oyo State. For this particular brand, limited published data on physical and insecticidal durability are available beyond what has been reported during WHO evaluation process [15]. In a retrospective study of DawaPlus^®^ 2.0 with adjustment for recall bias of nets obtained from the campaign in Nigeria, one of the sites was in Shinkafi LGA, Zamfara State, the LGA neighbouring Bakura LGA used in this study [12]. After 3 years 75% of the campaign nets had survived in serviceable condition corresponding to a median survival of 4.7 years (95% CI 4.4–5.1), very similar to the result in this study. In the other two sites median survival was 3.0 years in Nasarawa, central Nigeria and 4.5 years in Cross River in the South-south zone. In contrast, monitoring DawaPlus^®^ 2.0 in Mongala Province, northwest Democratic Republic of Congo (DRC) using the same methodology as this study resulted in functional survival of 17% after 31 months and median survival of 1.6 years (Mansiangi, manuscript submitted). This demonstrates the huge variation in durability the same LLIN brand can have depending where and by whom it is used. This finding is further supported by other studies using comparable methodology which found variations in physical durability of other brands and sites. For the polyethylene LLIN Olyset^®^ median survival estimates were reported ranging from 4.0 to 4.5 years in Kenya [17] to 2.5 years in Zambia [18] and 1.5–2.0 years in Benin [19]. Similarly, median survival results for the PermaNet^®^ 2.0 brand, a polyester LLIN similar to DawaPlus^®^, range from 3.5 years in Cambodia [20] to 2.5 years in Zambia [18] and around 1.0 years in Ethiopia [21].

The campaign nets monitored in this study were not all immediately used by the recipient households, an observation that has been previously documented [22]. But there were significant differences in the time it took until most of the nets were hanging ranging from 6 months to 2 years and in Oyo 45% of the cohort nets with a known outcome were never found hanging. This was on the one hand due to the availability of other nets in the household and on the other hand due to household decisions about whether to switch to the new nets immediately or continue using the older nets and keep the new ones for later or give them away to others. In Zamfara the former pattern was observed as all previously owned non-cohort nets had disappeared by 12 months follow-up and at the same time all-cause attrition of cohort nets remained low (Fig. 3). The opposite behaviour was seen in the Oyo site. Campaign nets were hung later and more were given away and this was due to a significant over-supply immediately following the campaign, with an average of 1.5 nets for every two household members, combined with continued influx of new nets throughout the study. This resulted in a high all-cause attrition of the cohort nets of 47% at 24 months compared to 39% in Ebonyi and 14% in Zamfara at the same time point. Such variation in the utilization of the cohort nets can be expected to have an impact on the measured physical survival and, indeed, this study showed a reduction in estimated median survival of 0.5–1.0 years if the risk of damage was considered to begin only when the net was first hung. For programmatic decisions on LLIN distribution strategies, i.e. the question when a next distribution campaign is needed, the intention-to-treat approach appears to be more adequate as it captures the real-life situation, which is the result of complex decisions by the household in the face of multiple sources of LLIN. In this case the survival estimate is not only the result of net use environment and behaviour but also the household LLIN supply situation. If, however, the purpose of the durability monitoring is focusing on the textile qualities of an LLIN brand, a per-protocol approach to analysis, i.e. including only nets ever hung, would be preferable provided net-use environment and behavioural aspects can be kept constant. This approach will still depend to some extent on the household’s decisions when new nets are obtained, but to a much lesser degree.

The drivers of better physical durability that were identified in this study using Cox proportionate hazard models were on the one hand general household characteristics such as a positive net care attitude and, closely associated with that, exposure to net care SBCC messages and, on the other hand, specific behaviours such as never leaving a net hanging loose over the sleeping place during the day and never cooking in the sleeping room. These have previously been found to have a positive impact in the Nigeria context [12, 13]. There is further evidence from Ethiopia that improved knowledge of net care and repair induces a more positive attitude [23]. A positive effect on physical durability was also seen for nets that had been exclusively used by adults (HR 0.66) and at the same time the presence of children under 5 years old had a detrimental effect (HR 1.42). Even if the evidence in this study for these factors was not very strong, it points to the importance of the net users and their use behaviour while under the net or getting in and out. The association of under-fives with damage had also been seen in the previous Nigeria durability studies where it was established that the larger the household, the more children under five there were, the higher the risks of damage to the nets [12, 13]. Closely related is the sleeping place over which the net is used. In this study chances of physical survival were somewhat poorer for nets used over a foam mattress compared to a bed frame and even poorer for use over a reed mat or the ground, but again the evidence in the multivariable model was not very strong, suggesting that it is not the sleeping place per se but certain sleeping places increase the risks if the user is not careful. Such similarly weak evidence of the negative impact on nets over reed mats was seen in Tanzania [24] and Zambia [18]. The sampled population in Zamfara had the most favourable constellation of household and net handling risk factors and this sufficiently explained the differences to the other two sites as shown by the observation that the site variable was no longer of any impact in the multivariable model. However, this was not the case for the peri-urban site in Oyo suggesting that some factors that could impact durability may not yet have been adequately captured. The socio-economic status of the household was not a significant factor in the final models in this study, but this does not mean that physical durability was not poorer in poorer households as most positive factors were associated with increased wealth and better education and hence poorer in poor households. This emphasizes the fact that poverty most of the time is just a proxy for the challenges to develop or adapt positive behaviours in difficult living conditions.

Of respondents in Zamfara, 95% stated that they used their nets equally in the rainy and dry seasons. Unfortunately, all surveys in this study were done in the rainy season so that dry season use could not be observed. But there is good evidence from other sources to suggest that in this dry savannah area with a short and heavy rain period and a long dry period LLIN use is seasonal [25, 26]. Therefore, the question arises whether a lower use rate of the cohort nets in the dry season could have contributed to the significantly better physical durability of the monitored LLIN in Zamfara. Since no data were collected in the dry season, this cannot be ruled out, but in the Cox proportionate hazard model the Zamfara site (brand) variable in the model had a hazard ratio of 0.91 compared to Ebonyi suggesting that in addition to the explanatory variables already in the model there was no difference between performance of the same brand in Zamfara and Ebonyi and that seasonality most likely did not play a significant role in the results. Furthermore, a more intensive and regular use of the cohort nets seen in Zamfara during the rainy season (see Table 3) may have compensated for a lower use in the dry season.

## Limitations

Some of the durability risk factors, such as net care and repair attitude as well as some of the outcomes, such as reason for net losses were based on the answers of the household members interviewed and therefore, are prone to recall or social desirability biases. With the prospective design there is also the potential for the Hawthorne effect, whereby being asked about net care and handling four times over the course of three years may have contributed to changes in behaviour. The standard durability monitoring approach tries to minimize this by conducting only four surveys versus every 6 months as had been done in some of the earlier studies. Furthermore, while the sample of the campaign net cohort was representative for the selected districts within each state, the district (LGA) selection was purposive and some caution is required when generalizing the findings to the states or even Nigeria as a whole.

## Conclusions

Durability monitoring of representative cohorts of campaign nets in Ebonyi, Zamfara and Oyo States of Nigeria showed significant differences in performance of the same LLIN brand in different locations with Zamfara performing significantly above expectations with an estimated median survival of 5.3 years compared to 3.3 and 3.2 for Ebonyi and Oyo, respectively. These differences could be attributed to different net-use environments and net handling behaviours. In addition, it was shown that under the intention-to-treat analysis survival was 0.5–1.0 years longer than if nets were only included once hung for the first time. This implies that if comparison of brand performance in the objective of durability monitoring, a per-protocol approach is preferable. All campaign net samples showed sufficient insecticidal effectiveness after three (Ebonyi and Zamfara) and two years (Oyo).

## Supplementary information

**Additional file 1.** Hypothetical loss functions with defined median survival. Presents detailed graph, formula and parameterization of hypothetical loss functions.

**Additional file 2.** Household characteristics. Contains table with demographic and socio-economic characteristics of sampled households.

**Additional file 3.** Survival functions intention to treat vs. per protocol. Presents graph of Kaplan–Meier survival function comparing sites separate for intention to treat and per protocol analysis.

## Data Availability

The datasets used and/or analysed during the current study are available from the corresponding author on reasonable request.

## Abbreviations

ANC: Ante-natal care
aHR: Adjusted hazard ratio
EPI: Expanded programme for immunization
HR: Hazard ratio
IPC: Inter-personal communication
ITN: Insecticide-treated net
LGA: Local Government Area
LLIN: Long-lasting insecticidal net
NMEP: National Malaria Elimination Programme
pHI: Proportionate hole index
SBCC: Social and behaviour change communication
SMEP: State Malaria Elimination Programme
