# Supplementary material for: Monitoring the physical and insecticidal durability of the long-lasting insecticidal net DawaPlus® 2.0 in three States in Nigeria
Source: Malar J. 2020 Mar 30;19:124. doi: 10.1186/s12936-020-03194-9 (PMC7106771; doi:10.1186/s12936-020-03194-9)
Supplement: Supplementary file 1 — Additional file 1. Hypothetical loss functions with defined median survival. Presents detailed graph, formula and parameterization of hypothetical loss functions. [file 12936_2020_3194_MOESM1_ESM.pdf]

Additional file 1

Hypothetical loss functions with defined median survival

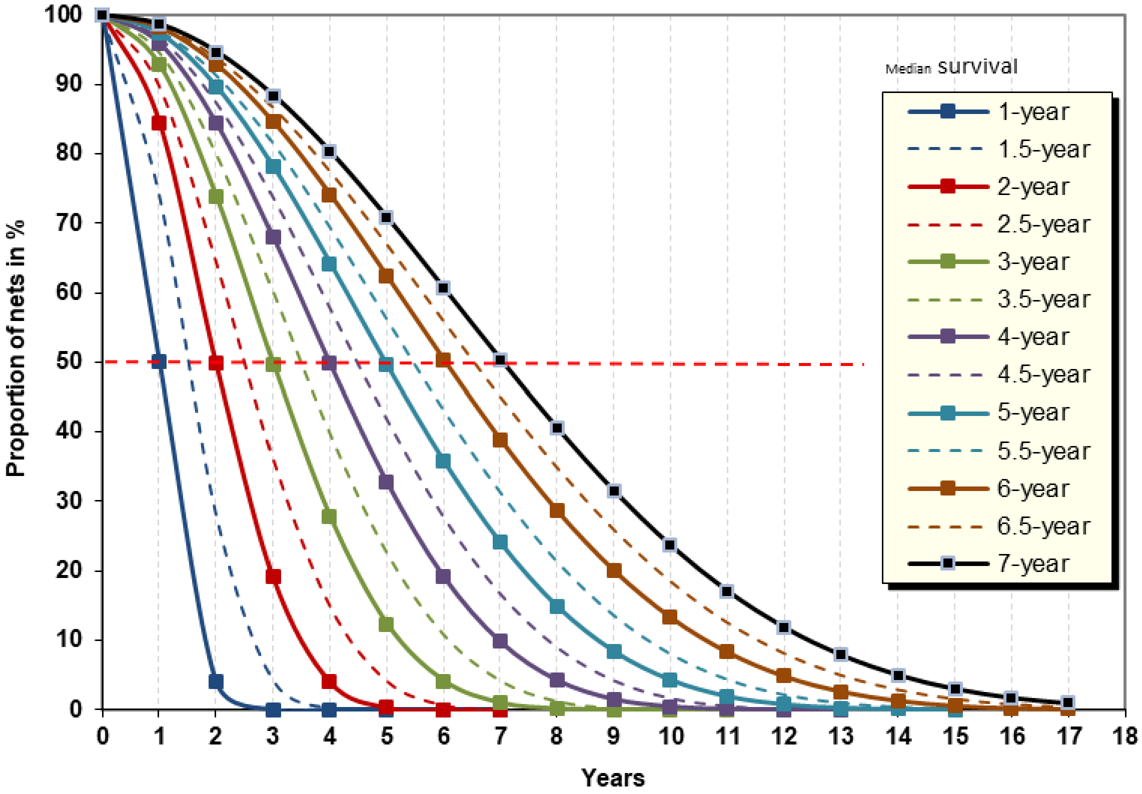

Formula for loss function

$$f(t) = \begin{cases} 100 \times \exp\left[k - \frac{k}{1-(t/L)^2}\right] & \text{if } t < L, \\ 0 & \text{if } t \geq L, \end{cases}$$

Values for t, L and k

t (years)	L	k
1.0	4.76	15.0
1.5	7.28	15.5
2.0	9.80	16.0
2.5	12.45	16.5
3.0	15.10	17.0
3.5	17.90	17.5
4.0	20.70	18.0
4.5	23.60	18.5
5.0	26.50	19.0
5.5	29.50	19.5
6.0	34.50	22.0
6.5	38.25	23.0
7.0	42.00	24.0
