# Supplementary material for: Monitoring the physical and insecticidal durability of the long-lasting insecticidal net DawaPlus® 2.0 in three States in Nigeria
Source: Malar J. 2020 Mar 30;19:124. doi: 10.1186/s12936-020-03194-9 (PMC7106771; doi:10.1186/s12936-020-03194-9)
Supplement: Supplementary file 2 — Additional file 2. Household characteristics. Contains table with demographic and socio-economic characteristics of sampled households. [file 12936_2020_3194_MOESM2_ESM.pdf]

Additional file 2

Household characteristics

Table: Household characteristics based on households that were seen at baseline and endline surveys.

Variable	Zamfara	Ebonyi	Oyo	P-value
	% (95% CI)*	% (95% CI)*	% (95% CI)*	for comparison between sites
**Demographic**				
Mean number of de-jure members	5.5 (5.1-6.0)	5.0 (4.5-5.4)	4.4 (4.0-4.8)	0.03
Mean age of head of household in years	41.6 (39.6-43.6)	51.1 (48.7-53.5)	50.0 (46.4-53.6)	0.09
Proportion of female headed households	1.9 (0.6-5.7)	8.5 (5.5-12.8)	22.6 (15.2-32.2)	<0.0001
Proportion of under-fives	22.4 (11.4-16.6)	13.8 (11.4-16.6)	10.3 (8.1-13.1)	<0.0001
Education of male heads of household				
Non-literate	40.6 (29.1-53.3)	34.5 (23.8-47.1)	30.2 (16.5-48.7)	
Primary	40.6 (28.5-54.0)	16.5 (10.5-25.0)	15.1 (9.2-23.7)	0.0003
Secondary	18.8 (12.6-27.0)	49.0 (36.1-62.0)	54.7 (37.5-70.9)	
**House characteristics**				
Improved roof materials	1.5 (0.5-4.6)	1.3 (0.3-5.4)	1.4 (0.5-3.7)	0.9
Improved wall materials	2.3 (1.0-5.1)	44.0 (28.0-61.0)	78.4 (59.2-90.1)	<0.0001
Improved floor materials	3.0 (0.8-10.9)	37.2 (22.9-54-2)	75.2 (54.7-88.4)	<0.0001
Cooking fuel kerosene or gas	0.0 (-.-)	3.8 (1.3-11.1)	51.4 (30.2-72.1)	<0.0001
**Water and sanitation**				
Access to safe water	97.4 (82.2-29.1)	14.1 (6.2-29.1)	92.7 (75.1-98.2)	<0.0001
Access to any latrine	100 (-.-)	72.7 (60.2-82.4)	66.5 (41.6-84.7)	0.004
Improved latrine or flush toilet	0.4 (0.1-2.8)	1.7 (0.7-4.2)	39.9 (20.0-63.8)	<0.0001
**Household assets**				
Any transport	72.0 (59.7-81.7)	82.9 (75.1-88.7)	41.3 (30.8-52.6)	<0.0001
Type of transport				
Bicycle	48.9 (38.8-59.0)	59.0 (42.6-73.6)	3.2 (1.2- 8.4)	<0.0001
Motorcycle	35.2 (25.7-46.1)	62.0 (56.5-67.1)	24.8 (14.3-39.4)	0.0001
Car	4.2 (1.9- 8.9)	7.7 (3.8-14.9)	19.3 (12.7-28.1)	0.0006
Owns farm land	99.2 (97.1-99.8)	85.0 (69.2-93.5)	47.7 (29.3-66.8)	<0.0001
Owns livestock	90.9 (82.5-95.5)	79.9 (69.5-87.4)	68.4 (55.2-79.1)	0.003
Type of livestock				
Chicken	65.9 (57.0-73.8)	71.4 (59.4-81.0)	54.1 (39.8-67.8)	0.1
Goats	79.2 (71.0-85.5)	51.7 (38.4-64.7)	37.2 (23.5-53.2)	<0.0001
Cows	34.5 (21.1-50.9)	13.7 (5.3-31.2)	0.9 (0.2- 3.4)	0.0005
Household items owned				
Radio	66.7 (58.4-74.0)	92.7 (88.8-95.3)	87.6 (78.6-93.2)	<0.0001
Television	9.8 (5.0-18.5)	41.0 (29.3-53.9)	61.0 (37.9-80.0)	0.0001
Refrigerator	0.8 (0.2- 3.0)	6.4 (2.8-14.2)	39.9 (23.1-59.7)	<0.0001
Fan	8.3 (4.1-16.4)	16.2 (9.4-26.7)	54.6 (31.5-75.9)	<0.0001
Iron	4.9 (2.2-10.9)	13.7 (6.8-25.5)	47.9 (27.8-68.7)	<0.0001
Any mobile phone	65.5 (54.5-75.1)	48.7 (33.9-63.7)	83.5 (66.1-92.9)	0.005
Smartphone or computer	0.8 (0.1- 5.6)	0.4 (0.1- 3.2)	31.2 (18.5-47.8)	<0.0001

*Unless otherwise indicated in column 1
