# Supplementary material for: Monitoring the physical and insecticidal durability of the long-lasting insecticidal net DawaPlus® 2.0 in three States in Nigeria
Source: Malar J. 2020 Mar 30;19:124. doi: 10.1186/s12936-020-03194-9 (PMC7106771; doi:10.1186/s12936-020-03194-9)

Additional File 3

Survival functions intention to treat vs. per-protocol

Kaplan-Meier survival functions of cohort nets comparing risk starting at distribution (intention to treat) versus starting at first hanging (per protocol)

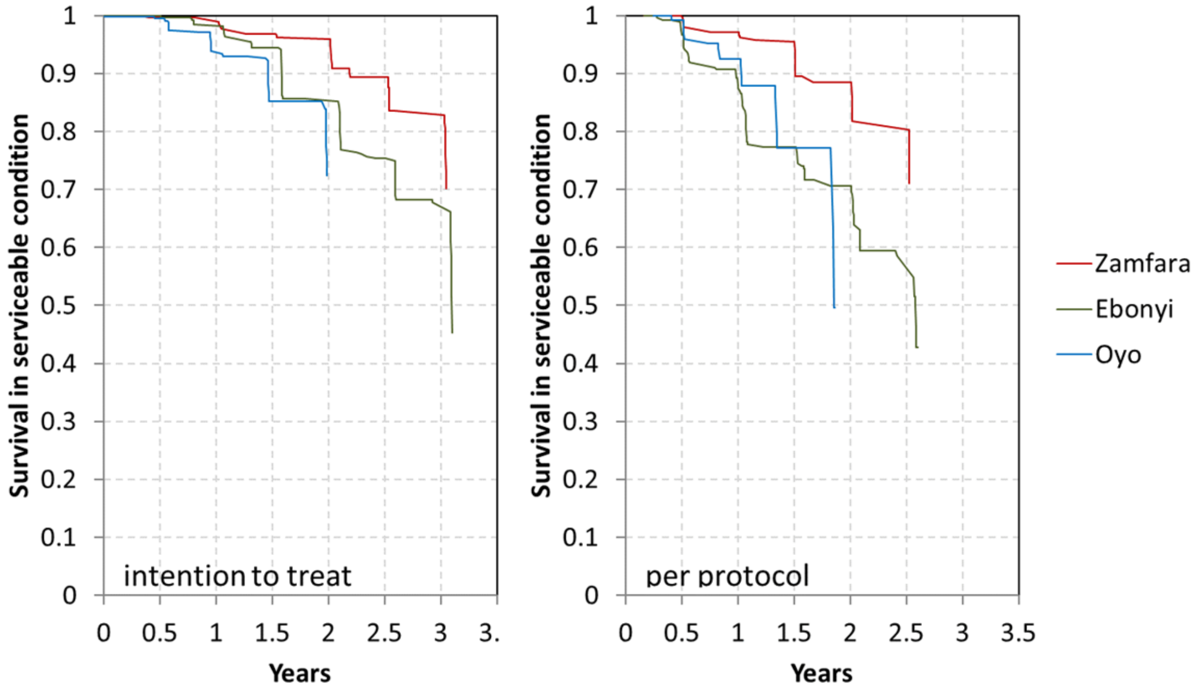

Supplement: Supplementary file 3 — Additional file 3. Survival functions intention to treat vs. per protocol. Presents graph of Kaplan–Meier survival function comparing sites separate for intention to treat and per protocol analysis. [file 12936_2020_3194_MOESM3_ESM.pdf]
